# Health-related quality of life and physical activity in children with Multiple Osteochondromas

**DOI:** 10.1016/j.jbo.2026.100759

**Published:** 2026-04-02

**Authors:** Ihsane Amajjar, Nienke W. Willigenburg, Benedikt G. Langenberg, S.John Ham, Rob J.E.M. Smeets

**Affiliations:** a Department of Rehabilitation Medicine, CAPHRI, Maastricht University, Maastricht, The Netherlands; b Department of Orthopaedic Surgery, OLVG, Amsterdam, The Netherlands; c Faculty of Health, Medicine and Life Sciences, Maastricht University, Maastricht, The Netherlands; d Pain in Motion International Research Group, www.paininmotion.be, Brussels, Belgium; e Clinics in Revalidatie (CIR), Eindhoven, The Netherlands

**Keywords:** Primary bone tumours, Quality of life, Physical activity, Exostoses, Multiple hereditary, Multiple osteochondromas

## Abstract

**Objectives:**

To evaluate health-related quality of life (HRQOL) and physical activity level (PAL) in children and adolescents with Multiple Osteochondromas (MO) and to identify factors associated with these outcomes.

**Study design:**

Cross-sectional survey of Dutch patients aged 4–18 years recruited through the national MO expertise center and the patient association. Validated, age-specific questionnaires assessed HRQOL (TAP/TAC/TAAQOL), PAL (Baecke), pain, functional disability, and psychosocial and sociodemographic variables. Multiple linear regression, guided by the International Classification of Functioning framework, examined associated variables with the mental and physical HRQOL components and with PAL.

**Results:**

A total of 134 participants (53% male; mean age ± SD, 11.4 ± 3.7 years) completed the survey. Compared with population norms, patients with MO scored significantly lower on physical HRQOL but not consistently lower on mental HRQOL. Significant gender differences emerged in specific HRQOL domains across multiple age groups. Mean PAL was also reduced (7.35 ± 1.01, p < 0.001) compared with healthy peers. Physical HRQOL (R^2^ = 0.727, p < 0.001) was negatively associated with pain and positively with externalizing behavior. The mental HRQOL model (R^2^ = 0.648, p < 0.001) showed the strongest negative associations with internalizing problems and functional disability. Having ≥ 5 surgeries was the only independent predictor of PAL, explaining 4.4% of its variance.

**Conclusions:**

The pediatric MO population reports significantly lower physical HRQOL and PAL, but not consistently lower mental HRQOL than healthy peers. Specific gender differences were observed across multiple domains. The associations identified in this study call for a multidisciplinary approach to MO management that emphasizes pain control, functional rehabilitation, and psychosocial support.

## Highlights

• Children with MO have lower physical HRQOL and physical activity levels than healthy peers.
• Mental and physical HRQOL are mostly reduced in adolescent girls.
• The interplay of physical, functional, and psychological factors in MO necessitates multidisciplinary care.

## 1 Introduction

Multiple Osteochondromas (MO) is a rare genetic disorder with numerous benign bone tumors, which can significantly impact daily lives [1]. These bone tumors, also called osteochondromas predominantly arise from the long bones’ metaphysis, and are caused by mutation of the EXT1 and EXT2 genes [2]. Osteochondromas develop during early childhood and typically stop growing after puberty [1]. The extended growth period in boys has been hypothesized to prolong disease progression and therefore severity [3].

MO can cause several complications such as compression of nerves, vessels, tendons, limb deformities and mobility restriction, thereby interfering with daily life and potentially reducing physical activity levels (PAL) [4], [5], [6], [7], [8]. D’Ambrosi et al. [4] reported lower sports activity in children affected by MO and Goud et al. [6] showed that 27% of children with MO stopped participating in sporting activities due to their disease. A recent Cochrane review (2023) on children with chronic disorders stated that challenges in exercising and participating in physical activities could result in missed school days and overall poor mental and physical health in adult life [9].

Most studies on health-related quality of life (HRQOL) in MO predominantly focus on adults and consistently report lower HRQOL compared to the general population [6], [10], [11], [12]. In a systematic review on adults with chronic pain disorders, PAL positively influenced both pain and HRQOL [13], suggesting that physical activity may also enhance HRQOL in MO. Although some studies reported on HRQOL in children with MO, the relationship between HRQOL and PAL remains unexplored. Boarini et al. (2024) observed that deformities and functional limitations are significant contributors to lower HRQOL in children with MO, but did not examine PAL as a contributing factor [10]. Understanding the interplay between HRQOL, PAL, and associated biopsychosocial factors in MO could help identify actionable targets for enhancing outcomes in children with MO.

Therefore, this study aims to: (1) evaluate HRQOL and PAL in the pediatric MO population; (2) compare HRQOL and PAL to gender- and age-matched healthy subjects and (3) identify factors associated with HRQOL and PAL respectively that could be used in future treatment strategies in this pediatric population.

## 2 Methods

### 2.1 Study design and participants

This prospective cross sectional survey study was performed from June 2019 to January 2022 in the Netherlands at the national MO expertise center (OLVG, Amsterdam). Children diagnosed with MO aged 4-18 years were invited to participate via the hospital database and via the Dutch patient association ‘HME-MO Vereniging Nederland’. Patients who could not speak Dutch were excluded. Participants completed validated and age-dependent questionnaires at their homes or during a routine outpatient visit. For children aged 4–7 years, a parent-proxy questionnaire was employed. Children aged 8–15 and adolescents aged 16–18 years completed the self-report versions of the survey. Participation was voluntary, and withdrawal was allowed at any time without explanation.

### 2.2 Data collection

**Fig. 1:**
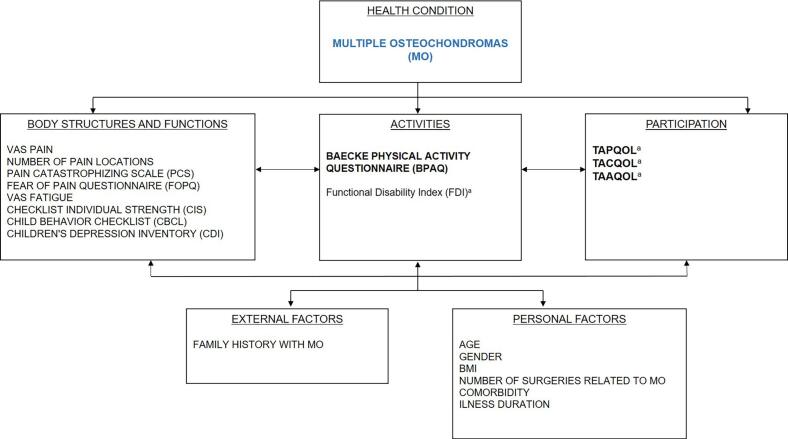
ICF model of the a priori selected explanatory variables for the dependent variables: the physical activity level (BPAQ) and health-related quality of life questionnaire for proxy parent (TAPQOL) children (TACQOL) and adolescents (TAAQOL) **^a^** Were not included in the regression model with the physical activity level (BPAQ) as dependent variable. The FDI measures disability at the activities level, equal to the ICF-level of the BPAQ. HRQOL, measured at the participation level, is assumed to be affected by the activities level and not the other way around.

Data collection was performed using the electronic data capture system Castor EDC [14], which reduced the risk of missing data by preventing participants to skip questions. The surveys were sent out via a secure email and consisted of several questionnaires with the main outcome parameters being HRQOL and PAL. The selection of questionnaires was guided by the International Classification of Functioning, Disability and Health (ICF) model (Fig. 1). The ICF is a theoretical framework developed by the World Health Organization used to define and measure disability and facilitate common language for discussing health issues [15]. Drawing from our study objectives, previous literature on MO and the ICF model, we formulated the following hypotheses: (1) children with MO have a lower PAL and HRQOL than healthy age and gender matched controls; (2) higher PAL, after controlling for other variables, is significantly associated with a higher HRQOL; (3) higher BMI, higher intensities of pain and fatigue, comorbidity, and the presence of psychological factors are negatively associated with PAL; (4) male gender, higher BMI, more surgical interventions, comorbidity, higher pain and fatigue levels, early illness onset, higher physical disability and the presence of psychological factors are negatively associated with HRQOL. Based on these hypotheses, the following questionnaires were selected.

### 2.3 Measures

The primary outcome measures for PAL and HRQOL are described as the dependent variables. The independent variables are those we hypothesized to be possibly associated with PAL and HRQOL.

#### 2.3.1 Dependent variables

PAL – Physical activity level over the past 12 months was measured with the Baecke Physical Activity Questionnaire (BPAQ), which consists of three domains: school, sport and leisure time. For the regression models the total score was used which has a range of 3–15 [16], [17]. Higher scores indicate higher levels of physical activity.

HRQOL – Health related quality of life was measured using the age-specific Dutch generic questionnaires developed by The TNO Institute of Prevention and Health and the Leiden University Hospital (TNO-AZL):

- TAPQOL (4-6 years): parent proxy measure consists of 12 scales: sleep, appetite, lung problems, stomach problems, skin problems, motor functioning, behavioral problems, social functioning, communication, positive mood anxiety, and liveliness. Total score: 0-86.
- TACQOL (6-16 years): self-report contains seven domains: physical complaints, motoric functioning, social functioning, autonomous functioning, cognitive functioning, positive emotions and negative emotions. Total score: 0-32.
- TAAQOL (16-18 years): self-report contains 12 domains: gross motor functioning, fine motor functioning, pain, sleeping, cognitive functioning, social functioning, daily activities, sexuality, vitality, positive, depressive, and aggressive emotions. Each domain scale ranges from 0 to 100.

A higher score on these HRQOL questionnaires indicates a better HRQOL. These questionnaires have been validated for the Dutch population and reference data from the general population, as reported in the questionnaires’ manuals, were used in this study [18], [19], [20], [21], [22], [23].

#### 2.3.2 Independent variables

Sociodemographic- and disease related factors were obtained as listed in Table 1.

**Table 1:** Patient characteristics, clinical data and independent variables.

	**N (Female: Male)**	**Total population** **Mean ± SD**	**Female Mean ± SD**	**Male** **Mean ± SD**
**Age**	134 (F:63 M:71)	11.4 ± 3.7	11.6 ± 3.5	11.2 ± 4.0
**BMI**	134	18.2 ± 3.2	17.7 ± 3.0	18.7 ± 3.3
**Illness duration**	134	7.5 ± 4.3	7.2 ± 4.3	7.8 ± 4.3
**Positive Family History MO, N (%)**	134	95 (70.9%)	46 (73%)	49 (69%)
**Comorbidities**	134	15 (11.2%)	8 (12.7%)	7 (9.9%)
**Deformities of the extremities, N (%)**	134	99 (73.9%)	55 (87%)	44 (62%)
**Number of surgical procedures**	134	2.4 ± 3.2	2.4 ± 3.2	2.3 ± 3.3
**Number of pain location sites**	134	3.0 ± 3.1	3.5 ± 3.5	2.6 ± 2.6
**Pain Catastrophizing Scale**	134	10.6 ± 9.2	10.0 ± 9.3	11.2 ± 9.0
**VAS Pain**	134	2.4 ± 2.4	2.6 ± 2.5	2.3 ± 2.3
**VAS Fatigue**	134	3.2 ± 2.6	3.7 ± 2.9	2.7 ± 2.3
**Checklist Individual Strength**	76 (F:38 M:38)^a^	66.3 ± 24.6	66.6 ± 27.0	66.1 ± 22.4
**Child Behavior Checklist**	127 (F:63 M:64)^a^			
**Total problems**		30.3 ± 18.4	30.6 ± 19.0	29.9 ± 17.9
Normal range		111 (87.4%)	56 (89%)	55 (85.9%)
Borderline		6 (4.7%)	4 (6.4%)	2 (3.1%)
Clinical range		10 (7.9%)	3 (5.8%)	7 (10.9%)
**Internalizing problems**		11.0 ± 9.1	11.9 ± 9.8	10.1 ± 8.4
Normal range		91 (71.7%)	46 (73%)	45 (70.3%)
Borderline		11 (8.7%)	7 (11.1%)	4 (6.3%)
Clinical range		25 (19.7%)	10 (15.9%)	15 (23.4%)
**Externalizing problems**		9.7 ± 6.7	9.3 ± 6.3	10.1 ± 7.0
Normal range		103 (81.1%)	53 (84.1%)	50 (78.1%)
Borderline		14 (11%)	8 (12.7%)	6 (9.4%)
Clinical range		10 (7.9%)	2 (3.2%)	8 (12.5%)
Functional Disability Inventory *	104 (F:50 M:54)^a^	7.6 ± 8.6	9.5 ± 10.1*	5.7 ± 6.5*
**Children's Depression Inventory**	104 a	6.9 ± 6.7	7.9 ± 8.0	5.9 ± 5.1
**Fear of Pain Questionnaire**	76 (F:38 M:38) a	26.8 ± 17.3	26.7 ± 18.9	27.0 ± 15.9

* p < 0.05 using independent samples *t*-test between genders.

a Participant numbers vary due to the use of age-specific questionnaires; see S1 Appendix for details.Abbreviations: VAS, visual analogue scale; F, female; M, male.

Pain was measured using different constructs:

• Pain intensity during the past week was measured by the Visual Analogue Scale (VAS-P; range 0-10).
• Amount of pain locations: patients could report pain on every possible body part.

Fatigue:

• Visual Analogue Scale (VAS-F; range 0-10); participants rated their fatigue over the past week.
• Checklist Individual Strength (CIS; range 20-140): contains four subscales: subjective experience of fatigue, concentration, motivation and physical activity. Higher scores indicate higher levels of fatigue [24], [25].

Psychosocial constructs:

• The Children’s Depression Inventory (CDI; range 0-54) was used to measure depression, with higher scores indicating higher levels of depressive symptoms [26], [27].
• The Child Behavior Checklist (CBCL) consists of a parent proxy form and child form with different measures on emotional and behavioral symptoms. The total scores and the subscales internalizing and externalizing problems were used [28].
• Functional Disability Inventory (FDI; range 0-60) was used to assess the perceived difficulty in performing activities in the domains recreation, school, home, and social interaction. Scores: minimal disability (0 –12), moderate disability (13–29), and severe disability (≥30) [29].
• The Pain Catastrophizing Scale (PCS; range 0–52) was used to assess pain catastrophizing. Higher scores indicate higher levels of catastrophic thinking [30].
• Fear of Pain Questionnaire (FOPQ; range 0-96) was used to assess pain related fears and avoidance. Higher scores indicate more fear and avoidance [31].

The questionnaires used are validated and age-specific, which explains why some data is not available for the younger age group in this study. An overview of the questionnaires and their applicability per age group is provided in S1 Appendix.

### 2.4 Statistical analysis

All analyses were performed in SPSS (version 27 for Windows, Chicago, IL, USA). Descriptive data were reported for patient characteristics and the prevalence rates of PAL, HRQOL and the psychosocial variables. Gender differences were analyzed using Chi-square or independent samples t-test. A one-sample t-test was used to compare scores of the dependent and independent variables to reference scores derived from normative data on healthy subjects, as reported in questionnaire manuals or published studies involving healthy peers. A Pearson correlation coefficient was computed to determine the association between HRQOL and PAL.

To study the association of the hypothesized variables with PAL and HRQOL, respectively, a purposeful selection method with two steps was used to develop three linear regression models. The dependent variables were BPAQ, HRQOL mental component scale (QOL-m), and HRQOL physical (QOL-p) component scale, all of which are continuous variables. To create the HRQOL mental and physical component scales a principal component analysis was performed to congregate the several scales in these two components. For this we used orthogonal rotation and standardized the scores to a mean of 50 (SD = 10), where scores above or below 50 represent above- or below-average functioning [32], [33], [34].

Independent variables for PAL and HRQOL were selected beforehand on the basis of the ICF model. The variables ‘number of surgical treatments’ and ‘pain location sites’ were each categorized into four groups: 0, 1–2, 3–4, and ≥5. These cut-offs were determined based on the distribution within our study population, aiming to create categories with approximately balanced group sizes. Those with a P value ≤ 0.25 in a univariable regression model were included in the multivariable model. We used backward selection with a significance threshold of p ≤ 0.05. The final model was checked for multicollinearity (Variation Inflation Factor, VIF<4 and Tolerance>0.1), normality of residuals and homoscedasticity [35], [36].

Based on a previous study conducted by Goud et al. [6], which included 99 children with MO, a minimum sample size of 100 participants was targeted. The actual obtained sample size (134 completed questionnaires) informed methodological choices in constructing the multivariable models and interpreting the results.

Ethical approval

The study was approved by the Medical research Ethics Committees United (MEC-U, Reference No. NL62341.100.17) and the Institutional Review Board ACWO-MEC (OLVG, Reference No. WO 17.171). Written informed consent was obtained from both participants and their guardian(s) for those under 16 years. Participants aged 16-18 years provided their own consent. In the Netherlands, individuals aged 16 years and older are legally entitled to provide informed consent independently, whereas those under 16 require consent from both the participant and their guardian(s).

## 3 Results

### 3.1 Patient characteristics

**Fig. 2:**
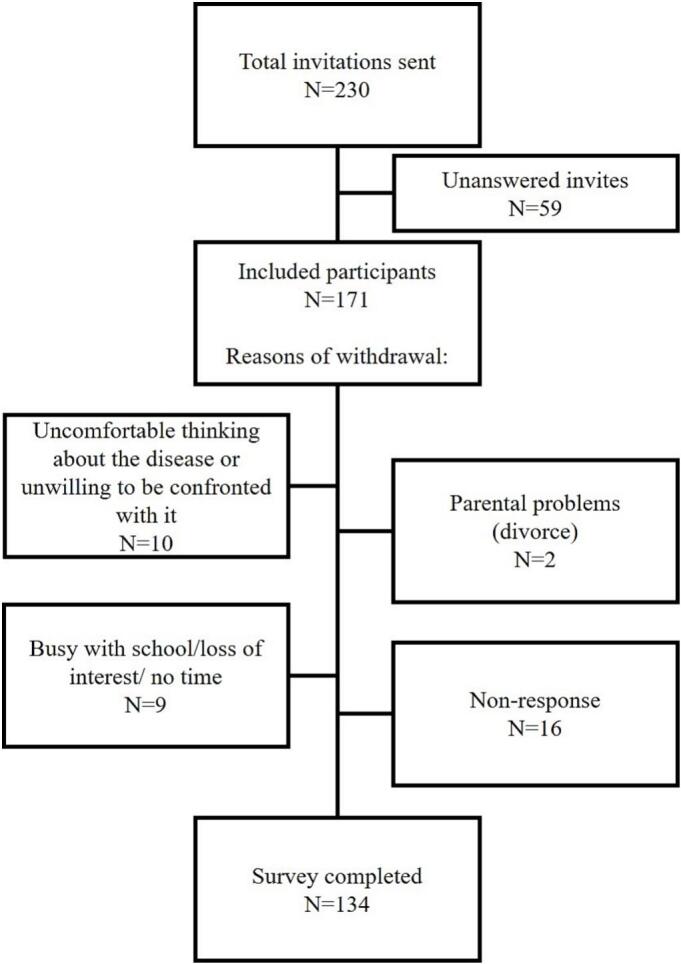
Included participant and outline of withdrawal reasons.

Of the 230 individuals invited, 171 provided informed consent and were enrolled. Among these, 134 completed the survey (response rate: 58%, completion rate 78%). The most frequently reported reason for withdrawal was participants' discomfort with being confronted by their disease. Fig. 2 outlines the additional reasons for withdrawal.

Patients’ characteristics are summarized in Table 1. Participants had a mean age of 11.4 ± 3.7, and 53% were male. A higher self-reported functional disability score was observed for female participants compared with male participants (p=0.027). Severe disability (FDI ≥30) was observed in 3.8% of the study population, while mild disability (12 < FDI <30) occurred in 17.3%. CDI scores showed that 7.7% of participants potentially had depression (CDI >19), and 9.7% reported thoughts of suicide. Compared to a healthy population (HP), the CDI score for the total MO group was significantly lower (mean HP 8.6 ± 6.2; p=0.004), as were the PCS (mean HP 16.8 ± 8.8; p<0.001), FDI (mean HP 8.2 ± 7.8; p=0.004), and FOPQ (mean HP 37.7 ± 16.1; p<0.001) [27], [30], [37], [38].

### 3.2 Health-related quality of life

The health-related quality of life (HRQOL) scores for the various age groups are presented in Table 2, Table 3, Table 4, Table 5.

**Table 2:** Health-related quality of life scores, parent proxy TAPQOL ages 4–5 years.

**TAPQOL scales 4**–**5 years** **(N = 7, M 7: F 0)**	**MO** **Mean ± SD**	**Healthy Dutch population** **Mean ± SD**	**p value**
**stomach problems**	77.4 ± 19.7	92.6 ± 13	0.037*
**skin problems**	91.7 ± 12.7	92.8 ± 10	0.822
**lung problems**	92.9 ± 18.9	97.2 ± 9	0.565
**sleeping**	81.3 ± 20.1	83.1 ± 17	0.816
**appetite**	96.4 ± 4.5	85.9 ± 12	<0.001*
**liveliness**	95.2 ± 8.1	98.1 ± 8	0.388
**positive mood**	100.0 ± 0.0	98.9 ± 6	0.016**
**problem behaviour**	78.6 ± 20.6	67.7 ± 15	0.212
**anxiety**	59.5 ± 25.2	79.2 ± 17	0.084
**social functioning**	97.6 ± 6.3	91.4 ± 15	0.040*
**motor functioning**	78.6 ± 27.5	98.8 ± 4	0.099
**communication**	95.5 ± 11.8	91.7 ± 10	0.423

* p < 0.05 one sample *t*-test ** Sign test.

Abbreviations: M: males, F: females, TAPQOL: TNO-AZL Preschool Children Quality of Life.

**Table 3:** Health-related quality of life scores, parent proxy TACQOL ages 6–7 years.

**TACQOL–PF scales 6**–**7 years**	**MO** **Mean ± SD** N = 23	**MO − Female Mean ± SD** N = 13	**MO − Male** **Mean ± SD** N = 10	**MO – Male vs. Female** **p-value**	**Healthy Dutch population** **Female Mean ± SD**	**MO vs Healthy Dutch** **Femalep-value**	**Healthy Dutch population** **Male** **Mean ± SD**	**MO vs Healthy Dutch** **Malep-value**
Body	26.7 ± 3.9	27.9 ± 2.3	25.0 ± 4.9	0.070	27.6 ± 3.4	0.642	28.5 ± 3.6	0.053
Motor	27.9 ± 5.2	30.1 ± 3.1	25.1 ± 6.1	0.036*	31.4 ± 1.5	0.150	31.1 ± 2.4	0.013*
Autonomy	30.1 ± 4.0	31.8 ± 0.4	28.0 ± 5.4	0.056	31.2 ± 2.7	<0.001*	31.1 ± 2.0	0.110
Cognition	29.3 ± 4.0	30.8 ± 1.6	27.3 ± 5.3	0.071	30.3 ± 2.7	0.313	29.3 ± 3.7	0.257
Social	29.4 ± 3.4	30.6 ± 1.9	27.9 ± 4.3	0.086	30.5 ± 1.8	0.764	30.1 ± 2.1	0.132
Emopos	14.5 ± 2.5	15.5 ± 0.7	13.2 ± 3.3	0.051	15.4 ± 1.1	0.433	15.1 ± 1.8	0.103
Emoneg	12.0 ± 2.4	12.3 ± 2.4	11.5 ± 2.5	0.441	11.8 ± 2.2	0.0474	11.5 ± 2.3	0.960

* p < 0.05 independent-samples *t*-test for male vs. female and one-sample *t*-test vs. healthy population.

Abbreviations**:** TACQOL:TNO-AZL Child Quality of Life Questionnaire. PF: parent form/proxy, Body: problems /limitations concerning general physical functioning/complaints. Motor: problems / limitations concerning motor functioning. Autonomy: problems / limitations concerning independent daily functioning. Cognition: problems / limitations concerning cognitive functioning and school performances. Social: problems / limitations in social contacts, with parents and peers. Emopos: the occurrence of positive moods. Emoneg: the occurrence of negative moods.

**Table 4:** Health-related quality of life scores, self-report TACQOL ages 8–15 years.

**TACQOL-CF scales8-15 years**	**MO Mean ± SD** N = 82	**MO − Female Mean ± SD** N = 39	**MO − Male** **Mean ± SD** N = 43	**MO − Male vs. Female** **p-value**	**Healthy Dutch population** **Female Mean ± SD**	**MO vs Healthy Dutch** **Femalep-value**	**Healthy Dutch population** **Male** **Mean ± SD**	**MO vs Healthy Dutch** **Malep-value**
Body	24.2 ± 6.3	23.9 ± 6.7	24.5 ± 5.9	0.658	25.2 ± 5.2	0.227	25.5 ± 4.8	0.237
Motor	24.9 ± 6.3	24.1 ± 6.7	25.7 ± 5.9	0.239	19.9 ± 3.4	<0.001*	30.1 ± 2.9	<0.001*
Autonomy	28.9 ± 4.7	28.1 ± 5.5	29.5 ± 3.7	0.167	31.2 ± 2.2	<0.001*	31.4 ± 1.5	0.002*
Cognition	26.3 ± 5.9	25.7 ± 6.5	26.9 ± 5.3	0.382	28.5 ± 4.2	0.010*	28.7 ± 3.6	0.031*
Social	28.8 ± 4.0	28.1 ± 5.1	29.5 ± 2.4	0.122	29.8 ± 2.8	0.046*	29.8 ± 2.5	0.337
Emopos	13.8 ± 2.9	13.5 ± 3.3	14.1 ± 2.4	0.332	13.8 ± 2.5	0.590	13.5 ± 2.5	0.104
Emoneg	11.6 ± 3.3	11.4 ± 3.3	11.9 ± 3.3	0.473	11.8 ± 2.6	0.437	11.7 ± 2.7	0.697

* p < 0.05 one-sample *t*-test MO vs. healthy population.

Abbreviations**:** CF: child form/self-report, HDP: Healthy Dutch population. Body: problems /limitations concerning general physical functioning/complaints. Motor: problems / limitations concerning motor functioning. Autonomy: problems / limitations concerning independent daily functioning. Cognition: problems / limitations concerning cognitive functioning and school performances. Social: problems / limitations in social contacts, with parents and peers. Emopos: the occurrence of positive moods. Emoneg: the occurrence of negative moods.

**Table 5:** Health-related quality of life scores, self-report TAAQOL ages 16–18 years.

**TAAQOL 16**–**18 years**	**MO** **Mean ± SD** N = 22	**MO − Female Mean ± SD** N = 11	**MO − Male** **Mean ± SD** N = 11	**MO Male vs. Female** **p-value**	**Healthy Dutch population** **Female** **Mean ± SD**	**MO vs Healthy Dutch** **Femalep-value**	**Healthy Dutch population** **Male** **Mean ± SD**	**MO vs Healthy Dutch** **Malep-value**
Gross motor functioning	67.3 ± 22.3	58.0 ± 17.0	76.7 ± 23.7	0.046 *	95.5 ± 11.3	<0.001*	96.9 ± 10.3	0.018*
Fine motor functioning	92.6 ± 10.9	90.9 ± 12.0	94.3 ± 9.9	0.475	98.9 ± 4.4	0.051	99.4 ± 4.2	0.118
Cognition	66.8 ± 31.4	48.9 ± 27.2	84.7 ± 25.1	0.004 *	88.3 ± 19.3	<0.001*	90.3 ± 16.6	0.472
Sleep	73.3 ± 31.6	65.3 ± 31.5	81.3 ± 31.0	0.247	77.6 ± 22.1	0.226	84.1 ± 19.3	0.767
Pain	62.8 ± 26.7	51.1 ± 21.3	74.4 ± 27.3	0.037 *	80.7 ± 18.3	<0.001*	85.4 ± 16.7	0.212
Social contacts	86.1 ± 22.0	76.1 ± 27.4	96.0 ± 7.0	0.039 *	89.0 ± 15.8	0.150	89.0 ± 15.0	0.008*
Daily activities	67.3 ± 31.6	56.3 ± 26.5	78.4 ± 33.5	0.101	89.5 ± 18.4	0.002*	91.1 ± 16.4	0.237
Sexuality	96.6 ± 16.0	93.2 ± 22.6	100 ± 0.0	#	91.0 ± 18.9	0.756	89.2 ± 21.9	#
Vitality	51.1 ± 28.7	43.2 ± 21.4	59.1 ± 33.6	0.203	68.8 ± 20.4	0.003*	76.0 ± 17.7	0.126
Happiness	68.2 ± 22.2	66.7 ± 24.2	69.7 ± 21.2	0.757	70.5 ± 19.8	0.610	69.6 ± 19.4	0.988
Depressive mood	70.5 ± 23.7	62.1 ± 24.3	78.8 ± 20.9	0.100	80.9 ± 17.5	0.028*	85.6 ± 15.4	0.304
Anger	79.8 ± 23.4	76.8 ± 26.0	82.8 ± 21.3	0.557	89.4 ± 14.5	0.139	89.9 ± 14.4	0.297

* p < 0.05 one-sample *t*-test MO vs. healthy population.

Abbreviations: TAAQOL:TNO-AZL Adult Quality of Life.

# One-sample *t*-test not performed due to zero standard deviation in the male MO group.

In our group of 4-5-year-old children with MO (n=7 males), outcomes were largely similar to healthy peers. Any observed differences could be due to chance, given the small sample size in this specific age group.

Among children with MO aged 6–7 years (n=23), parents reported HRQOL scores similar to healthy peers in most domains However, boys with MO reported significantly lower motor skills compared to girls with MO (MD=4.98, p=0.036) and compared with age-matched peers from the healthy Dutch reference population (p=0.013). Girls with MO scored lower in autonomy skills compared to their healthy counterparts (p <0.001).

For participants with MO aged 8–15 years (n=82), no gender differences emerged within the group. However, compared with age and gender-matched healthy peers, girls and boys with MO exhibited significantly lower motor function, greater challenges with autonomy and daily activities, impaired cognition and school performance. Specifically, among girls with MO significantly increased social difficulties were reported compared with their healthy peers (Table 4).

In the oldest group (16–18 years, n=22), girls with MO reported lower gross motor function, decreased cognition, more pain, and greater social difficulties compared with boys. Girls with MO, compared with healthy peers, scored significantly lower in gross motor functioning, cognition, pain, daily activities, vitality, and depressive symptoms. In contrast, boys with MO showed significantly lower scores compared to healthy peers in only two domains: gross motor functioning and social contacts. (Table 5).

As described in the Methods, we performed a principal component analysis to group the multiple HRQOL domains into two components—Physical and Mental Quality of Life. Boys and girls with MO had consistently lower physical HRQOL than healthy peers. Mental HRQOL was only significantly reduced in adolescent girls with MO. (Table 6).

**Table 6:** Principal component analysis – physical and mental component scale.

**HRQOL**		**MO** **Mean ± SD**	**Healthy Dutch population** **Mean ± SD**	**MO vs. Healthy Dutch population** **p- value**	**Female** **Mean ± SD**	**Male** **Mean ± SD**	**Male vs. Female** **p-value**
**Physical Component Scale**	**TACQOL** **N = 105** **(age 8**–**15)**	38.9 ± 16.32	50.2 ± 9.5	<0.001*	36.7 ± 18.2	41.0 ± 14.3	0.236
	**TAAQOL** **N = 22** **(age 16**–**18)**	26.6 ± 19.9	50.0 ± 10.1	<0.001*	18.3 ± 16.9	34.8 ± 20.0	0.049*
**Mental Component Scale**	**TACQOL** **N = 105** **(age 8**–**15)**	49.5 ± 12.0	50.6 ± 8.8	0.393	47.9 ± 2.4	51.0 ± 8.9	0.249
	**TAAQOL** **N = 22** **(age 16**–**18)**	42.9 ± 13.3	50.1 ± 9.1	0.019*	36.8 ± 13.9	49.0 ± 9.8	0.014*

* p < 0.05 one-sample *t*-test MO vs. healthy population.

Abbreviations: HRQOL, health related quality of life; TACQOL, TNO AZL Children's Quality of Life; TAAQOL TNO-AZL Adolescent Quality of Life.

For the below presented multiple regression models, the independent variables included and excluded following the univariable analyses (first step) are presented in S2 Appendix.

Table 7 presents the final regression model for the mental component of HRQOL, with four independent variables that explain 64.8% of the variance. CBCL internalizing problems showed the strongest unique contribution (19.6%), indicating that higher internalizing scores corresponded to lower mental HRQOL. Older age was associated with lower mental HRQOL (3.3%), while greater functional disability (FDI) accounted for 3.4% of the variance and negatively influenced HRQOL. Higher BMI was positively associated with mental HRQOL (2.7%).

**Table 7:** Summary of backward multiple linear regression analysis for Mental Health-Related Quality of Life, Physical Health-Related Quality of Life, and physical activity level as dependent variables.

**Independent variable**	**Unstandardized ß**	**ß**	**95%CI**	**Adj. R^2^**	**P value**	**sr^2^**	**VIF**
	**Model: Mental Health-Related Quality of Life (M−HRQOL)**						
				0.648	<0.001		
CBCL-Internalizing problems	−0.813	−0.597	−1.06; −0.56		<0.001	0.196	1.813
FDI	−0.374	−0.243	−0.65; −0.10		0.009	0.034	1.735
Age	−1.477	−0.208	−2.60; −0.36		0.010	0.033	1.277
BMI	0.852	0.182	0.14; 1.57		0.020	0.027	1.241
	**Model: Physical Health-Related Quality of Life (P-HRQOL)**						
				0.727	<0.001		
VAS pain	−2.658	−0.360	−3.79; −1.53		<0.001	0.080	1.623
CBCL-Externalizing problems	0.726	0.218	0.21; 1.25		0.007	0.028	1.683
CBCL-Internalizing problems	−0.630	−0.347	−0.96; −0.30		<0.001	0.051	2.351
FDI	−0.857	−0.419	−1.22; −0.49		<0.001	0.078	2.239
	**Model: physical activity level (Baecke)**						
				0.044	0.037		
Five or more surgeries	0.528	0.239	0.03;1.02		0.037	0.057	1.000

Abbreviations: Adj. R^2^ = adjusted explained variance, sr^2^ = squared semi-partial correlation, VIF = variance inflation factor, CBCL=Child Behavior Checklist, FDI = Functional Disability Inventory, BMI = Body Mass Index.

The multivariable regression model for physical HRQOL is also shown in table 7. The final model accounted for 72.7% of the variance with four independent variables. VAS pain demonstrated the strongest unique contribution (8.0%), indicating that higher pain scores were associated with poorer physical HRQOL. Greater functional disability (FDI) and elevated CBCL internalizing problems uniquely explained 7.8% and 5.1% of the variance, respectively, reflecting adverse associations with physical HRQOL. In contrast, CBCL externalizing problems contributed 2.8% of the variance and was positively associated with physical HRQOL.

### 3.3 Physical activity level

No significant differences emerged in PAL among boys and girls with MO. However, their scores were significantly lower than the reference value for healthy Dutch children (MD=1.38, p<0.001; Table 8).

**Table 8:** Physical Activity Levels − Baecke.

	**MO** **Mean ± SD**	**Healthy controls**^a^ **Mean ± SD**	**p-value** **MO vs Healthy controls**	**95%CI** **MO vs Healthy controls**
**Baecke total**	7.35 ± 1.01	8.73 ± 1.19	<0.001	−1.66; −1.02
Females	7.31 ± 1.05	8.62 ± 1.15	<0.001	−1.65; −0.96
Males	7.39 ± 0.98	8.88 ± 1.21	<0.001	−1.81; −1.17
**School**	2.32 ± 0.49	2.75 ± 0.34	<0.001	−0.58; −0.33
Females	2.34 ± 0.58	2.72 ± 0.32	<0.001	−0.57; −0.19
Males	2.29 ± 0.38	2.80 ± 0.37	<0.001	−0.63; −0.38
**Leisure**	2.70 ± 0.55	3.04 ± 0.62	<0.001	−0.58; −0.20
Females	2.74 ± 0.53	3.06 ± 0.61	<0.001	−0.49; −0.14
Males	2.65 ± 0.57	3.02 ± 0.62	<0.001	−0.56; −0.18
**Sports**	2.34 ± 0.54	2.97 ± 0.67	<0.001	−0.69; −0.34
Females	2.23 ± 0.54	2.86 ± 0.64	<0.001	−0.81; −0.45
Males	2.45 ± 0.53	3.11 ± 0.67	<0.001	−0.83; −0.48

a Reference scores from ten Velde et al., 2021 (The Netherlands)[17].

^a^ Reference scores from ten Velde et al., 2021 (The Netherlands) [17].

In Table 7, the final multivariable regression model for PAL is presented. After backward selection of the independent variables, only having undergone five or more surgeries showed to be significantly and positively associated with PAL, which explained 4.4% of the total variance. There were no significant associations between PAL and either mental (MCS: r = 0.11, 95% CI [–0.12, 0.33], p = 0.333) or physical (PCS: r = 0.09, 95% CI [–0.14, 0.31], p = 0.427) HRQOL.

## 4 Discussion

In this cross-sectional study conducted in the Netherlands, patients with MO reported significantly lower physical HRQOL and PAL compared to the healthy general population. Children (8-15 years old) with MO had comparable mental HRQOL to that of healthy children, while adolescent girls with MO (16-18 years old) scored significantly lower than adolescent boys with MO and healthy peers. Contrary to our expectations, our findings revealed no significant association between PAL and HRQOL, indicating that other factors may have greater influence on these patients’ quality of life. Several relevant determinants of HRQOL were identified, collectively explaining substantial variance in both the mental (64.8%) and physical (72.7%) components of HRQOL. Specifically, greater pain intensity, increased internalizing behaviors, older age, and higher functional disability independently predicted lower HRQOL scores. Conversely, externalizing behaviors and a higher BMI were related to higher HRQOL scores. Additionally, undergoing more than five surgical procedures was associated with higher PAL, potentially reflecting improved functional capacity in more severely affected patients.

To the best of our knowledge, this study represents the largest pediatric population with MO to date investigating both HRQOL and PAL. The HRQOL of patients in MO has previously been evaluated primarily among adult populations, and only few studies have addressed this topic in pediatric population [6], [10], [11], [12]. The lower HRQOL scores observed in our study, compared with reference values from healthy peers, are consistent with previous reports. A more detailed exploration of HRQOL domains revealed that a majority of participants experienced notable impairments in motor skills, autonomy, school performance, and social interactions, highlighting the substantial impact of MO on various facets of daily life among children. Several associations with HRQOL identified in this study align with previous literature concerning MO as well as those with other chronic conditions. Internalizing behaviors, greater functional disability, and older age were each independently associated with lower mental HRQOL in our cohort. To date, behavioral problems and coping strategies in MO populations remained uncharacterized. However, a systematic review in pediatric Crohn’s disease demonstrated that internalizing symptoms predict poorer quality of life, increased functional disability, pain-related distress, and school difficulties [39]. These effects may stem from inward-focused emotional distress, which amplifies symptom perception and undermines adaptive coping. Age-related declines in HRQOL have likewise been reported [10], [12], and mirror our findings; such declines likely reflect the cumulative burden of disease—repeated surgical interventions, progressive deformities, and heightened social awareness with age. Conversely, a higher BMI was associated with higher HRQOL. Boarini and colleagues (2024) found no significant association between BMI and HRQOL in their MO cohort [10]. However, Chen and colleagues (2024) described a consistent U-shaped relation between BMI and mental health in the general pediatric population [40], indicating that both lower and higher BMI can be associated with poor mental HRQOL. In our cohort, the mean BMI was 18.7 ± 3.29, and the majority of the patients fell below accepted pediatric obesity thresholds. These findings suggests that (reducing) BMI is not likely a relevant treatment target in children and adolescents with MO.

The physical component of HRQOL was negatively associated with functional disability and pain, consistent with prior studies [10], [11], [12]. However, in contrast to literature in general pediatric populations, where higher externalizing behaviors typically is associated with poorer HRQOL [41], we observed a positive association between externalizing scores and physical HRQOL. In MO, externalizing traits, such as assertiveness and impulsiveness, may prompt children to engage more actively in physical activities, thereby enhancing perceived well-being; alternatively, these traits could lead them to overestimate their capabilities and under-report limitations, inflating HRQOL scores.

Contrary to our hypothesis, no significant association was found between PAL and HRQOL. Literature on PAL in MO is scarce, and no prior study explored its relationship with HRQOL [4], [6], [11], [12]. Our results contrasts with literature on other chronic diseases [13], [42], [43], yet aligns with a 2023 Cochrane review that found insufficient evidence for a quality-of-life benefit from physical activity in children and adolescents with chronic musculoskeletal pain [9]. Psychosocial factors may exert a stronger influence on PAL in MO, masking any direct link with HRQOL. Functional-disability scores in our cohort were lower compared with population norms. A plausible explanation is a response-shift phenomenon, indicating that affected children rate their PAL and HRQOL differently, because they adapt to their condition. A concerning finding, however, is that 9.7 % of participants reported suicidal thoughts. Although mean Child Depression Inventory scores in our cohort were lower than normative values, the observed prevalence of suicidal ideation underscores the need for routine screening. Van Tilburg reported that adolescents with chronic pain have a higher risk of suicidal ideation, only partly explained by comorbid depression [44]. In 2021, 8.5% of the general pediatric population in the Netherlands reported suicidal ideation [45], based on data collected during the COVID-19 pandemic, a period that coincided with the present study. It is plausible that the broader psychological impact of the pandemic contributed to this finding. Several studies have highlighted the negative effects of COVID-19 on the mental health of children and adolescents [46].

Consistent with previous studies, children with MO demonstrated significantly reduced PAL across school, leisure, and sports domains compared to healthy peers. D'Ambrosi et al. (2018) and Goud et al. (2012) reported similar findings, emphasizing that lower PAL is linked to pain, functional limitations, and psychosocial factors [4], [6]. Our study extends these observations by identifying surgical history as a significant determinant of PAL, with children who had undergone five or more surgeries reporting higher PAL. Contrary to certain adult studies linking multiple surgeries with higher pain and poorer outcomes [5], [12], [47], our results suggest that a higher number of surgeries may correlate with better PAL in childhood. This could reflect differences in surgical indications and recovery protocols in pediatric populations, or a tendency for children with severe deformities (prompting repeated surgery) to gain more functional benefit postoperatively. Our data underscore the need for further longitudinal or interventional studies to discern whether early, targeted surgery can sustainably enhance activity and function.

Previous studies reported on associations between more severe skeletal phenotype or unfavorable EXT1 genotype with worse outcomes in males,[1], [48] whereas others showed poorer health-related outcomes in females [12], [47]. In our cohort, boys aged 6–7 years showed lower motor-skill scores, possibly because girls generally attain fine-motor milestones earlier, giving them an early functional advantage. From 8 to 15 years the gap closed, but after 15 years girls demonstrated inferior motor function and greater social difficulties. This adolescent shift may relate to a worse phenotype or perhaps heightened body-image concerns, stronger peer comparisons, and higher pain sensitivity in females, all of which can amplify functional and social limitations [49]. Consistent with D’Ambrosi et al. [12], adolescent and adult women with MO also reported greater psychological distress than men. However, no gender differences were observed in PAL. These findings underscore the need for age- and gender-specific monitoring and support throughout growth.

Certain limitations must be acknowledged when interpreting these findings. First, due to the cross-sectional design, causal relationships cannot be established. Second, selection bias remains a concern, as recruitment through the expertise center and patient association could have favored symptomatic or more severely affected individuals; to mitigate this risk, we also invited patients without scheduled orthopedic visits, drawing from the hospital registry.

In this study, only painful lesion sites were assessed rather than all lesions, providing a limited indication of disease severity. A more comprehensive characterization of the total number and anatomical distribution of osteochondromas would have better captured disease severity, but this would require whole-body imaging, which was not available for the study population. Moreover, disease severity in Multiple Osteochondromas cannot be captured by lesion count alone, as classifications such as the IOR classification also incorporate skeletal deformities and functional limitations to assess phenotypic disease burden [50]. Future studies should therefore integrate phenotypic severity with clinical outcomes, including quality of life and physical activity.

The interpretation of the associated variable ‘age’ in the regression model should be approached with caution because of differences between age-specific questionnaires. This is an inherent limitation, as the use of distinct, age-appropriate instruments is necessary for valid assessment but precludes direct comparability across age groups. Therefore, the potential confounding effect of the questionnaires themselves cannot be entirely ruled out. Physical activity was captured only with self-report instruments; the absence of motor control tests, accelerometry or gait analysis limits objectivity. Recall bias is a known limitation of survey studies but was minimized through the use of a prospective study design and predominantly questions about the patients’ current status. In addition, the rarity of MO precluded a formal a-priori power calculation, so subtle associations could have been missed.

Balanced against these constraints, the study enrolled the largest pediatric MO cohort published to date and applied a prospective design with age-validated scales for HRQOL, psychosocial factors, and PAL, thereby enhancing measurement reliability.

### 4.1 Clinical implications

The strong influence of internalizing distress on HRQOL underscores the need for routine psychosocial screening in MO. Instruments such as the Child Behavior Checklist or age-appropriate depression inventories can identify at-risk patients early and prompt timely referral to mental-health services, potentially preventing long-term deterioration in quality of life.

Because functional disability, pain, and psychological distress are interrelated, management should be multidisciplinary. Conservative strategies, including individualized physiotherapy, structured pain-management protocols, and psychological support, are likely to yield greater HRQOL gains than exercise prescriptions alone. Pain control, continued monitoring of emotional health, and support for functional independence should therefore be prioritized.

Surgical procedures can improve mobility and activity when clearly justified, but their potential benefits must be balanced against operative risks. Prospective studies that measure PAL and HRQOL before and after surgery are needed to define optimal indications and expected outcomes. Similarly, trials of tailored exercise programs or combined physical-and-psychological rehabilitation will clarify whether structured interventions enhance activity engagement and overall well-being. Finally, longitudinal research should examine the long-term impact of PAL on HRQOL and disease progression in MO. Given the chronic nature of MO, the recommended multidisciplinary approach should be explicitly integrated into structured transition plans from pediatric to adult healthcare services. This transition period poses significant challenges for adolescents with MO and requires dedicated attention to safeguard their health-related quality of life and prevent potential deterioration in their well-being [51].

## 5 Conclusion

In conclusion, this study shows that children with MO experience significantly lower physical HRQOL and PAL compared with healthy peers. Mental HRQOL was similar to healthy pears in boys and young girls, but reduced in adolescent girls with MO. Greater pain intensity, functional disability, internalizing behavior, and older age are strongly associated with lower HRQOL, whereas externalizing traits and a higher BMI appear to be linked with better HRQOL. Physical activity level itself is not an independent determinant of HRQOL, and the unexpectedly higher PAL in surgically treated children suggests that well-selected procedures may restore function. The 9.7% prevalence of suicidal thoughts highlights the need for systematic psychosocial surveillance. Together, these findings advocate a multidisciplinary care model that prioritizes pain control, functional rehabilitation, and routine mental-health screening. Future prospective studies—using objective activity monitors and pre-/post-operative follow-up—should clarify causal pathways and test combined physical and psychological interventions aimed at improving the quality of care in MO.

## CRediT authorship contribution statement

**Ihsane Amajjar:** Writing – review & editing, Writing – original draft, Project administration, Methodology, Investigation, Funding acquisition, Formal analysis, Data curation, Conceptualization. **Nienke W. Willigenburg:** Writing – review & editing, Supervision, Methodology, Conceptualization. **Benedikt G. Langenberg:** Formal analysis. **S.John Ham:** Writing – review & editing, Supervision, Methodology, Conceptualization. **Rob J.E.M. Smeets:** Writing – review & editing, Supervision, Methodology, Conceptualization.

## Declaration of competing interest

The authors declare that they have no known competing financial interests or personal relationships that could have appeared to influence the work reported in this paper.
