# Supplementary material for: Health-related quality of life and physical activity in children with Multiple Osteochondromas
Source: J Bone Oncol. 2026 Apr 2;58:100759. doi: 10.1016/j.jbo.2026.100759 (PMC13087577; doi:10.1016/j.jbo.2026.100759)
Supplement: Supplementary Data 2 [file mmc2.docx]

# S2 Appendix. Univariable linear regression analysis (first step of 2-step model)

**Univariable linear regression analysis for HRQOL-mental component**

- A two-step approach was applied. First, independent variables based on the ICF model (Figure 1) were analysed using univariable linear regression analysis. Variables with p ≤ 0.25 were selected for inclusion in the second step, which is presented in the main text.
- The tables below display the results of the first step of the analysis.

|  | **Dependent variable – mental component HRQOL** | | | |
| --- | --- | --- | --- | --- |
| **Independent variables** | **Standardized coefficients** | **Adjusted R^2^** | **95% CI** | **P- value** |
| VAS – pain | -0.348 | 0.114 | -0.492;-0.184 | <0.001 |
| Nr. pain location |  |  |  |  |
| 1-2 | 0.204 | 0.034 | 0.031;0.366 | 0.021 |
| 3-4 | 0.072 | -0.003 | -0.103;0.243 | 0.421 |
| ≥5 | -0.418 | 0.168 | -0.552;-0.263 | <0.001 |
| VAS – fatigue | -0.471 | 0.215 | -0.596;-0.323 | <0.001 |
| CIS – total score | -0.595 | 0.345 | -0.723;-0.427 | <0.001 |
| PCS | -0.415 | 0.165 | -0.549;-0.259 | <0.001 |
| FOPQ | -0.435 | 0.194 | -0.616;-0.253 | <0.001 |
| CBCL total problems | -0.646 | 0.412 | -0.737;-0.531 | <0.001 |
| CBCL Externalizing | -0.530 | 0.275 | -0.644;-0.392 | <0.001 |
| CBCL Internalizing | -0.741 | 0.546 | -0.811;-0.651 | <0.001 |
| FDI | -0.578 | 0.328 | -0.693;-0.434 | <0.001 |
| CDI | -0.698 | 0.482 | -0.785;-0.584 | <0.001 |
| Baecke | 0.113 | -0.001 | -0.116;0.330 | 0.333 |
| Age | -0.301 | 0.083 | -0.451;-0.134 | <0.001 |
| Gender | -0.108 | 0.004 | -0.277;0.068 | 0.227 |
| BMI | -0.182 | 0.025 | -0.345;-0.008 | 0.041 |
| Comorbidity | -0.128 | 0.009 | -0.296;0.047 | 0.152 |
| Positive family history MO | 0.142 | 0.012 | -0.033;0.308 | 0.112 |
| Nr. Surgical procedures |  |  |  |  |
| 1-2 | -0.018 | -0.008 | -0.192;0.157 | 0.840 |
| 3-4 | 0.129 | 0.009 | -0.047;0.296 | 0.150 |
| ≥5 | -0.271 | 0.066 | -0.426;-0.102 | 0.002 |
| Illness duration | -0.085 | -0.001 | -0.256;0.090 | 0.341 |

*Abbreviations: HRQOL – Health-Related Quality of Life; VAS – Visual Analogue Scale; CIS – Checklist Individual Strength; PCS – Pain Catastrophizing Scale; FOPQ – Fear of Pain Questionnaire; CBCL – Child Behavior Checklist; FDI – Functional Disability Inventory; CDI – Children's Depression Inventory; BMI – Body Mass Index*

|  | **Dependent variable – physical component HRQOL** | | | |
| --- | --- | --- | --- | --- |
| **Independent variables** | **Standardized coefficients** | **Adjusted R^2^** | **95% CI** | **P- value** |
| VAS – pain | -0.698 | 0.483 | -0.778;-0.597 | <0.001 |
| Nr. pain location |  |  |  |  |
| 1-2 | 0.301 | 0.084 | 0.134;0.452 | <0.001 |
| 3-4 | -0.053 | -0.005 | -0.225;0.123 | 0.556 |
| ≥5 | -0.568 | 0.317 | -0.675;-0.437 | <0.001 |
| VAS – fatigue | -0.713 | 0.505 | -0.790;-0.616 | <0.001 |
| CIS – total score | -0.570 | 0.315 | -0.705;-0.395 | <0.001 |
| PCS | -0.386 | 0.143 | -0.525;-0.228 | <0.001 |
| FOPQ | -0.548 | 0.291 | -0.688;-0.368 | <0.001 |
| CBCL total problems | -0.560 | 0.308 | -0.669;-0.428 | <0.001 |
| CBCL Externalizing | -0.332 | 0.103 | -0.479;-0.168 | <0.001 |
| CBCL Internalizing | -0.611 | 0.368 | -0.710;0.489 | <0.001 |
| FDI | -0.757 | 0.569 | -0.829;-0.661 | <0.001 |
| CDI | -0.485 | 0.228 | -0.620;-0.323 | <0.001 |
| Baecke | 0.093 | -0.005 | -0.136;0.311 | 0.427 |
| Age | -0.370 | 0.130 | -0.512;-0.210 | <0.001 |
| Gender | -0.084 | -0.001 | -0.254;0.092 | 0.348 |
| BMI | -0.271 | 0.066 | -0.425;-0.101 | 0.002 |
| Comorbidity | -0.139 | 0.012 | -0.306;0.036 | 0.118 |
| Positive family history MO | 0.033 | -0.007 | -0.142;0.206 | 0.711 |
| Nr. Surgical procedures |  |  |  |  |
| 1-2 | -0.050 | -0.006 | -0.222;0.126 | 0.579 |
| 3-4 | 0.019 | -0.008 | -0.155;0.193 | 0.828 |
| ≥5 | -0.322 | 0.096 | -0.470;-0.156 | <0.001 |
| Illness duration | -0.313 | 0.091 | -0.462;-0.146 | <0.001 |

**Univariable linear regression analysis for HRQOL – physical component**

*Abbreviations: HRQOL – Health-Related Quality of Life; VAS – Visual Analogue Scale; CIS – Checklist Individual Strength; PCS – Pain Catastrophizing Scale; FOPQ – Fear of Pain Questionnaire; CBCL – Child Behavior Checklist; FDI – Functional Disability Inventory; CDI – Children's Depression Inventory; BMI – Body Mass Index*

|  | **Dependent variable – physical activity - Baecke** | | | |
| --- | --- | --- | --- | --- |
| **Independent variables** | **Standardized coefficients** | **Adjusted R^2^** | **95% CI** | **P- value** |
| HRQOL-M | 0.113 | -0.001 | -0.116;0.330 | 0.333 |
| HRQOL-P | 0.093 | -0.005 | -0.136;0.311 | 0.427 |
| VAS – pain | 0.004 | -0.014 | -0.222;0.229 | 0.975 |
| Nr. pain location |  |  |  |  |
| 1-2 | 0.186 | 0.002 | -0.041;0.395 | 0.107 |
| 3-4 | -0.053 | -0.11 | -0.275;0.175 | 0.650 |
| ≥5 | -0.030 | -0.013 | -0.254;0.197 | 0.798 |
| VAS – fatigue | -0.063 | -0.10 | -0.284;0.165 | 0.592 |
| CIS – total score | -0.117 | 0.000 | -0.334;0.111 | 0.312 |
| PCS | 0.157 | 0.011 | -0.071;0.369 | 0.176 |
| FOPQ | -0.022 | -0.013 | -0.246;0.204 | 0.849 |
| CBCL total problems | -0.019 | -0.013 | -0.243;0.208 | 0.872 |
| CBCL Externalizing | 0.011 | -0.013 | -0.215;0.236 | 0.925 |
| CBCL Internalizing | -0.028 | -0.013 | -0.252;0.199 | 0.812 |
| CDI | -0.087 | -0.006 | -0.307;0.141 | 0.453 |
| Age | 0.073 | -0.008 | -0.155;0.293 | 0.534 |
| Gender | -0.039 | -0.012 | -0.263;0.188 | 0.735 |
| BMI | 0.098 | -0.004 | -0.130;0.317 | 0.398 |
| Comorbidity | 0.100 | -0.003 | -0.128;0.318 | 0.391 |
| Positive family history MO | -0.007 | -0.13 | -0.232;0.218 | 0.949 |
| Nr. Surgical procedures |  |  |  |  |
| 1-2 | -0.122 | 0.002 | -0.338;0.106 | 0.293 |
| 3-4 | -0.028 | -0.013 | -0.252;0.198 | 0.808 |
| ≥5 | 0.239 | 0.044 | 0.014;0.441 | 0.037 |
| Illness duration | -0.026 | -0.13 | -0.250;0.201 | 0.827 |

**Univariable linear regression analysis for physical activity – Baecke**

*Abbreviations: HRQOL – Health-Related Quality of Life; VAS – Visual Analogue Scale; CIS – Checklist Individual Strength; PCS – Pain Catastrophizing Scale; FOPQ – Fear of Pain Questionnaire; CBCL – Child Behavior Checklist; FDI – Functional Disability Inventory; CDI – Children's Depression Inventory; BMI – Body Mass Index*
